# Cyclotron production of ^43^Sc for PET imaging

**DOI:** 10.1186/s40658-015-0136-x

**Published:** 2015-12-04

**Authors:** Rafał Walczak, Seweryn Krajewski, Katarzyna Szkliniarz, Mateusz Sitarz, Kamel Abbas, Jarosław Choiński, Andrzej Jakubowski, Jerzy Jastrzębski, Agnieszka Majkowska, Federica Simonelli, Anna Stolarz, Agnieszka Trzcińska, Wiktor Zipper, Aleksander Bilewicz

**Affiliations:** Institute of Nuclear Chemistry and Technology, Dorodna 16, 03-195 Warsaw, Poland; Department of Nuclear Physics, University of Silesia, Katowice, Poland; Heavy Ion Laboratory, University of Warsaw, Warsaw, Poland; Nuclear Security Unit, Joint Research Centre, Institute for Transuranium Elements, European Commission, Ispra, Italy; Nuclear Decommissioning Unit, Joint Research Centre, Ispra Site Management Directorate, European Commission, Ispra, Italy; Synektik S.A., Research and Development Center, Warsaw, Poland

**Keywords:** Scandium-43, Cyclotron production, Calcium target, Alpha irradiation, PET radiopharmaceuticals

## Abstract

**Background:**

Recently, significant interest in ^44^Sc as a tracer for positron emission tomography (PET) imaging has been observed. Unfortunately, the co-emission by ^44^Sc of high-energy γ rays (*E*_γ_ = 1157, 1499 keV) causes a dangerous increase of the radiation dose to the patients and clinical staff. However, it is possible to produce another radionuclide of scandium—^43^Sc—having properties similar to ^44^Sc but is characterized by much lower energy of the concurrent gamma emissions. This work presents the production route of ^43^Sc by α irradiation of natural calcium, its separation and purification processes, and the labeling of [DOTA,Tyr3] octreotate (DOTATATE) bioconjugate.

**Methods:**

Natural CaCO_3_ and enriched [^40^Ca]CaCO_3_ were irradiated with alpha particles for 1 h in an energy range of 14.8–30 MeV at a beam current of 0.5 or 0.25 μA. In order to find the optimum method for the separation of ^43^Sc from irradiated calcium targets, three processes previously developed for ^44^Sc were tested. Radiolabeling experiments were performed with DOTATATE radiobioconjugate, and the stability of the obtained ^43^Sc-DOTATATE was tested in human serum.

**Results:**

Studies of ^nat^CaCO_3_ target irradiation by alpha particles show that the optimum alpha particle energies are in the range of 24–27 MeV, giving 102 MBq/μA/h of ^43^Sc radioactivity which creates the opportunity to produce several GBq of ^43^Sc. The separation experiments performed indicate that, as with ^44^Sc, due to the simplicity of the operations and because of the chemical purity of the ^43^Sc obtained, the best separation process is when UTEVA resin is used. The DOTATATE conjugate was labeled by the obtained ^43^Sc with a yield >98 % at elevated temperature.

**Conclusions:**

Tens of GBq activities of ^43^Sc of high radionuclidic purity can be obtainable for clinical applications by irradiation of natural calcium with an alpha beam.

## Background

Radionuclide therapy of somatostatin overexpressing tumors is currently being performed with DOTA conjugated to somatostatin analogs (DOTATOC and DOTATATE) labeled with high- and medium-energy β^−^ emitters: ^90^Y or ^177^Lu, respectively [1, 2]. Many clinical studies have also shown that ^68^Ga-labeled somatostatin analogs are relevant positron emission tomography tracers for imaging such tumors and their metastases [3]. DOTATOC labeled with ^68^Ga showed high binding affinity to the human somatostatin receptor subtype 2, improving tumor imaging capabilities and offering the possibility of low dose imaging followed by higher dose treatment. However, the half-life of ^68^Ga (*T*_1*/*2_ = 67*.*71 min) may limit the spectrum of clinical applications of ^68^Ga-labeled radiopharmaceuticals. Furthermore, the relatively high cost of the generators and perhaps more importantly the requirement for postelution purification and concentration of ^68^Ga solution to small volume make this isotope of limited utility in clinical applications [4]. Therefore, the use of radionuclides of extended physical half-life is currently being reconsidered.

An alternative could be cyclotron-produced ^64^Cu (*T*_1/2_ = 12.7 h) which has been applied in a large number of preclinical and clinical positron emission tomography (PET) studies [5]. The longer half-life offers the possibility to label bigger molecules like mAB fragments and to use ^64^Cu radiopharmaceuticals in hospitals without cyclotrons and radiopharmaceutical units. However, ^64^Cu exists in three oxidation states and forms unstable in vivo chelate complexes. In addition, ^64^Cu presents a relatively low positron branching ratio (17.6 %) and high co-emission of β^−^ particles (39 % branching ratio) which significantly contribute to an additional patient dose.

In 2010, the ^44^Sc radionuclide was proposed by Roesch as a potential alternative to ^68^Ga in clinical PET diagnosis [6, 7]. ^44^Sc decays by the emission of low-energy positrons *E*_β+_, with a half-life of *T*_1/2_ = 3.97 h, which is almost four times longer than that of ^68^Ga. ^44^Sc can be obtained from the ^44^Ti/^44^Sc generator [8] or produced in the ^44^Ca(p,n)^44^Sc reaction on small- or medium-sized medical cyclotrons that currently supply ^18^F to hospitals [9–14]. These properties make it highly attractive for clinical PET applications because they enable transportation of ^44^Sc-labeled radiopharmaceuticals to hospitals that are located several hundred kilometers away from the radiopharmaceutical production site. Moreover, it was found that Sc^3+^ likewise Y^3+^ and Lu^3+^, forms in aqueous solutions of DOTA complexes with the same coordination sphere (CN = 8) and with similar stability constants [15], whereas the relatively small Ga^3+^ forms octahedral complexes. As a result, the chemical properties of ^44^Sc-labeled DOTA conjugates are almost the same as those of the ^90^Y- and ^177^Lu-labeled versions; therefore, we can presume that ^44^Sc-DOTA bioconjugates will demonstrate similar properties in vivo (i.e., receptor affinity, kidney clearance) to the ^177^Lu- and ^90^Y-conjugates currently applied in therapy.

It is also important to mention that the other scandium radioisotope, i.e., ^47^Sc (*T*_1/2_ = 3.4 days, *E*_β−(av)_ = 162 keV, main *E*_γ_ = 159.4 keV, *I* = 68.3 %) is a promising low-energy β^−^ emitter for targeted radiotherapy [16–19]; thus, the β^+^-emitting ^44^Sc with the β^−^-emitting ^47^Sc represent an ideal theranostic pair as mentioned above regarding ^64^Cu.

However, the co-emission of high-energy γ rays (*E*_γ_ = 1157, 1499 keV) has to be taken into consideration with regard to the radiation dose to the patients and clinical staff. Also, co-production of longer lived ^44m^Sc (*T*_1/2_ = 58.6 h) increases the radiation dose.

In our work, we propose to use another radionuclide of scandium, i.e., ^43^Sc, which shows properties similar to ^44^Sc, but with much lower energy concurrent gamma emissions (Table 1). ^43^Sc can be produced either by the ^43^Ca(p,n), or ^42^Ca(d,n) reactions, but unfortunately, the cost of enriched calcium targets is prohibitive. A more promising method of ^43^Sc production is alpha irradiation of a natural calcium target via the ^40^Ca(α,p) and ^40^Ca(α,n) channels. The possibility of ^43^Sc production by this route has already been mentioned in four conference communications. The availability of cyclotrons with intense α beams is limited; however, with a near-to-4-h half-life and predicted production cross-section approaching 1 b [20], the potential exists for regional distribution following mass production at a single cyclotron unit. The aim of this study was to investigate the possibility of ^43^Sc production at an accelerator, allowing its use for preclinical and clinical PET imaging.

**Table 1:** Comparison of ^68^Ga, ^44^Sc, and ^43^Sc nuclear properties

	^68^Ga	^44^Sc	^43^Sc
*T*_1/2_ [h]	1.14	3.92	3.89
β^+^ [% emission]	89	95	88
*E*_β+max_ [MeV]	1.90	1.47	1.20
*E*_γ_ [keV]	1077 (3 %)	1157.0 (99 %)	372.8 (23 %)
Generator production	^68^Ge (*T*_1/2_ = 270.8 days)/^68^Ga	^44^Ti (*T*_1/2_ = 60.4 y)/^44^ Sc	^–^
Cyclotron production	^68^Zn(p,n)^68^Ga	^44^Ca(p,n)^44^Sc	^43^Ca(p,n)^43^Sc
		^44^Ca(d,2n)^44^Sc	^42^Ca(d,n)^43^Sc
			^nat^Ca(α,p)^43^Sc
			^nat^Ca(α,n)^43^Ti→^43^Sc

## Methods

### Chemicals and reagents

NaOH micropills and acetic acid were purchased from POCH S.A. Gliwice. Ammonia (ammonium hydroxide solution 25 %), citric acid, and ammonium carbonate were purchased from Sigma Aldrich. N,N,N′,N′-tetra-n-octyldiglycolamide (DGA) 50–100 mesh and UTEVA 100–200 mesh resins were purchased from Eichrom, USA; Chelex 100 resin (Na^+^ form, mesh size 100–200) was purchased from Bio-Rad, USA; and DOWEX 50 × 8 resin (hydrogen form, 200–400 mesh) was purchased from Fluka Analytical, Germany. [DOTA,Tyr3] octreotate (DOTATATE) 95 % purity (HPLC) was purchased from piChem (Graz, Austria). All chemicals were of analytical grade and were used without further purification.

Natural CaCO_3_ of chemical purity >99.999 purchased from Sigma Aldrich and enriched [^40^Ca]CaCO_3_ (99.99 %) purchased from Isoflex (USA) were used as target materials. The isotopic composition of enriched ^40^Ca was 99.99 % of ^40^Ca and 0.01% of ^44^Ca, while the amounts of other calcium isotopes were below 0.01 %.

### Irradiation of ^nat^CaCO_3_ and [^40^Ca]CaCO_3_ targets

Irradiations of natural targets were performed using the Scanditronix MC 40 cyclotron at the European Commission’s Joint Research Centre (Ispra, Italy). Irradiations of enriched [^40^Ca]CaCO_3_ targets were performed using the Warsaw Heavy Ion Cyclotron operating at the Heavy Ion Laboratory of the University of Warsaw. The Ispra cyclotron is capable of accelerating positive ions such as protons, deuterons, and alphas to variable energies. The Warsaw machine accelerates heavy ions from +He up to Ar with energies from 2 up to about 8 AMeV. For irradiation at the Ispra cyclotron, the target material was wrapped in an aluminum foil of a 25-μm thickness. The samples were irradiated in aluminum capsules with an inner diameter of 10 mm. Each target capsule was inserted in a holder that allowed direct water cooling from both the rear and the front sides. In the Warsaw cyclotron, targets in the form of pellets bundled in thin aluminum foils produced from CaCO_3_ powder using a hydraulic press were irradiated with an internal α-particle beam. Al energy degraders were used when alpha particle energies lower than maximal were necessary.

In order to optimize the yield of ^43^Sc production by the ^40^Ca(α,p)^43^Sc and ^nat^Ca(α,n)^43^Ti→^43^Sc nuclear reactions, ~100-mg ^nat^CaCO_3_ samples (target thickness ~375 μm) were irradiated for 28–34 min by an alpha beam of 13–25 MeV on the target with an alpha current of 0.5 pμA at the Scanditronix MC 40 cyclotron.

Enriched [^40^Ca]CaCO_3_ targets of ~100 mg were irradiated for 30 min by an alpha beam of 20 MeV with an alpha current of 0.25 pμA (He^+^) at the Warsaw Heavy Ion Cyclotron.

### Measurement of radioactivity

The absolute radioactivity of ^43^Sc and other obtained radionuclides was measured by γ-spectrometry using two high-purity germanium (HPGe) detectors. The detectors were energy and efficiency calibrated in different geometries using certified standard radioactive sources (ENEA Italy, DAMRI and CERCA France). The gamma-ray spectrum analysis software package Genie 2000 (CANBERA, USA) was used to collect the data. The γ-ray peak at 372.8 keV was chosen for ^43^Sc detection, and the peaks at 1157.00, 271.24, and 159.38 keV were chosen to detect ^44^Sc, ^44m^Sc, and ^47^Sc, respectively. Three peaks at 983.52, 1037.52, 1312.10 keV were used to quantify yields of ^48^Sc [21]. The uncertainty of all the determined activities was below 1 %.

### Separation of ^43^Sc from the target

In order to find the optimal method for the separation of ^43^Sc from the irradiated calcium targets, three procedures were tested:

In the first method, described by Valdovinos et al. [22], the irradiated ^nat^CaCO_3_ target was dissolved in 1 ml of 9 M HCl solution. The dissolved target solution was passed through a column containing 50 mg of UTEVA resin, and after adsorption of ^43^Sc, the column was washed with 5 ml of 9 M HCl. The scandium radionuclides were eluted with a 400-μl portion of H_2_O.

The second method, reported in the paper by Mueller et al. [10], consists of dissolving the CaCO_3_ targets in 3 M HCl and adsorption of scandium radionuclides in a column filled with 70 mg of DGA. The adsorbed ^43^Sc was eluted from the DGA resin with HCl (0.1 M, 2–3 ml). Afterwards, the acidic ^43^Sc solution was loaded on a second column filled with 100 mg of cation exchange resin DOWEX 50 (hydrogen form, 200–400 mesh). Finally, ^43^Sc was eluted using 1 M ammonium acetate adjusted to pH = 4 using HCl solution.

The third method, developed by our group [9], consisted of dissolution of the target in 1 M HCl and adsorption of ^43^Sc on chelating ion exchange resin Chelex 100 of bed size 0.8 × 4.0 cm and conditioned with 5 ml of 1 M HCl. After adsorption of ^43^Sc and Ca^2+^, the column was washed with 30 ml of 0.01 M HCl in order to remove Ca^2+^. The scandium radionuclides were then eluted with 1 M HCl in 0.5-ml fractions.

### Radiolabeling and stability studies of DOTATATE conjugate

DOTATATE, octreotate-somatostatin analog conjugated to DOTA chelator, was labeled with the obtained ^43^Sc using 10, 15, and 25 nmol of the peptide. The most active fraction of ^43^Sc solution was combined with 0.2 ml of 0.2 M sodium acetate buffer (pH = 6) containing 14, 21, or 36 μl of the peptide (0.7 nmol μl^−1^) in the buffer. The solution was next heated for 25 min at 95 °C in a water bath. Product formation and reaction yields were estimated by instant thin-layer chromatography (ITLC) using Silica gel 60 TLC plates (Merck). A 0.1 M citric buffer of pH = 5.4 was used as the eluent. Of the solution, 10 μl was dropped on the ITLC strip. Free ^43^Sc moved with the front boundary of the solution whereas the labeled bioconjugate remained at the starting point. The labeling yield defined as the percentage of ^43^Sc radioactivity complexed by DOTATATE to the starting activity was calculated as the ratio of the activity of the strip application part to the whole strip activity.

The stability of the labeled DOTATATE in human serum was assessed by adding 20 μl of the radioconjugate solution to 500 μl of the human serum. The mixture was incubated at 37 °C, and the stability was measured by taking aliquots of the human serum solutions at different times and measuring the liberated scandium radionuclide by ITLC analysis.

## Results and discussion

### Optimization of ^43^Sc cyclotron production

The first step towards developing a simple, fast, and inexpensive method of ^43^Sc-DOTATATE production is the optimization of the cyclotron production parameters. For this purpose, we measured the radioactivity yield as a function of alpha particle energy on target. The results are presented in Table 2. The analysis of the results obtained shows that the optimum on-target α particle energies are in the range of 24–26.5 MeV, which is a little higher than that predicted by Levkovskij [23].

**Table 2:**
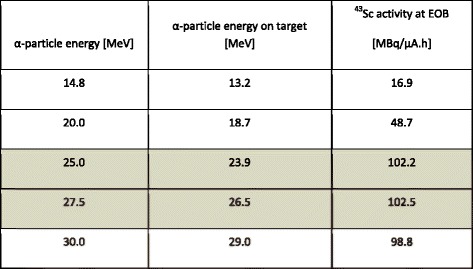
Activity of ^43^Sc as a function of alpha energy on target. The optimum proton beam energy is highlighted in gray

The ^43^Sc activity obtained after irradiation of a ~100 mg ^nat^CaCO_3_ target for 34 min with a 25-MeV alpha particle beam of a 0.5-μA beam current was about 29 MBq. The produced activity can be increased by extending the irradiation time and using a higher beam current, as in the case of ^44^Sc production by proton irradiation of a [^44^Ca]CaCO_3_ target [24]. Extrapolating to an irradiation time of 4 h at 20 μA, the end of bombardment (EOB) yields are expected to approach 5.7 GBq, enough for the preparation of more than 25 patient doses. Of course, the proposed 40-fold scale-up of the current will bring challenging problems with heat dissipation from the CaCO_3_ target, which can be solved by the use of a metallic Ca target previously tested by Severin et al. [11] for high-current proton irradiation.

Production of ^43^Sc is accompanied by small co-production of other scandium radionuclides such as ^44^Sc, ^44m^Sc, ^46^Sc, and ^47^Sc (Table 3). The scandium radioisotopes ^44g^Sc and ^44m^Sc were synthesized from ^42^Ca present in the natural target (0.65 %) via the (α,pn) reaction. ^46^Sc and ^47^Sc were produced from the 2 % of ^44^Ca and the 0.13 % of ^43^Ca components of the natural calcium in (α,pn) and (α,p) reactions, respectively.

**Table 3:** The radioactive impurities produced during 34 min

Radioisotope	Gamma energy [keV]	Decay mode	Half-life	Nuclear reaction	Activity EOB [kBq]
^44g^Sc	1157.00^a^	β^−^	3.93 h	^42^Ca(α,pn)	32
^44m^Sc	271.24^a^	β^−^	58.6 h	^42^Ca(α,pn)	3
^46^Sc	889.25^a^	β^−^	83.79 days	^43^Ca(α,p)	0.26
	1120.51			^44^Ca(α,pn)	
^47^Sc	159.38^a^	β^−^	3.35 days	^44^Ca(α,p)	5

Irradiation of a 100-mg target by a 0.5-μA alpha beam current of 25 MeV. The radioactivity of the produced ^43^Sc is equal to 28.9 MBq

^a^Energies of the γ-photon used for activity measurement of the radionuclide

As shown in Table 3, the only significant contaminant is the ^44g^Sc (0.011 %). From the point of view of possible applications of ^43^Sc in nuclear medicine contamination of the ^43^Sc product by ^44g^Sc is insignificant due to the similarity of both radioisotopes. Complete elimination of the ^43^Sc impurities is possible by using an isotopically enriched (and inexpensive) [^40^Ca]CaCO_3_ target (1.5 USD/mg). Irradiation of such a target (composed of 99.99 % ^40^Ca + 0.01 % ^44^Ca) with 20-MeV alpha particles results in a level of all impurities below 1.5 × 10^−5^ % of the ^43^Sc radioactivity, even 20 h after the EOB.

### Separation of ^43^Sc from the target

Three methods previously developed for ^44^Sc production were tested for the separation of ^43^Sc from the natural Ca target. One method was based on the application of chelating resin and was developed in our group [9], and in the other two methods, the extraction resins developed by Valdovinos et al. [13] and Mueller et al. [10] were used. These methods were compared with respect to ^43^Sc recovery, the volume and composition of the ^43^Sc fraction (Table 4), and the possibility of separation from metallic impurities which could negatively affect the effectiveness of ^43^Sc bioconjugate labeling.

**Table 4:** Comparison of radiochemical separation procedures used for the isolation of ^43^Sc from calcium matrix

Method	^43^Sc recovery (%)	Volume of ^43^Sc eluated (ml)	Composition of the obtained solution
Chelex 100	85	0.4	1M HCl
DGA + DOWEX 50	87	0.65	1 M NH_4_Ac/HCl (pH = 4)
UTEVA	80	0.4	0,8 M HCl

All separation procedures studied are fast and simple. In the case of Chelex 100 and UTEVA resins, the target dissolution and separation of ^43^Sc were performed in 30 min and the two-step separation process DGA + Dowex 50 in 45 min. All methods render possible the effective separation of ^43^Sc from calcium. The efficiency of the separation methods is consistent with the previously reported procedures for separation of ^44^Sc from enriched and natural targets [9, 10, 13].

High chemical purity of the final ^43^Sc fraction is important, since the presence of other metals may interact with the DOTA chelator. The most dangerous is Fe^3+^ for which the log of the stability constant with the DOTA ligand is 29.4 [25] which is greater than that for Sc^3+^ (log *K* = 27 [15]). Influence of other possible impurities like Zn^2+^and Co^2+^ is negligible due to the much lower stability constants of their DOTA complexes (log *K*_Zn_ = 19.3, log *K*_Co_ = 19.3 [25]. Fe concentration, measured with the ICP-MS technique in the dissolved calcium target in HCl solutions, varied between 58 and 87 ppm. After the separation processes, the total level of Fe in the ^43^Sc samples decreased to 10.50 ppm for separation with Chelex resin, 0.56 ppm for DGA + DOWEX 50 and <0.001 ppm for UTEVA. The amount of Ca^2+^ in the ^43^Sc fractions was less than 1 ppm.

Two additional important factors in the labeling processes are the volume of the ^43^Sc fraction and the composition of the eluate. In the three methods tested here, the volumes of eluates containing more than 80 % of ^43^Sc which was used for reprocessing varied between 0.4 and 0.65 ml. The best composition of the eluate was obtained using the tandem of DGA and Dowex 50 resins where the eluate containing ammonium acetate buffer can be used directly to label DOTA or DTPA bioconjugates. The acidic eluates from Chelex 100 and UTEVA resins need neutralization. From the experiments performed, it can be concluded that, as with ^44^Sc [13], due to the simplicity of the operations, the best methods for isolation of ^43^Sc from the target material are procedures in which Chelex 100 or UTEVA resins are used. In respect to the chemical purity of the obtained ^43^Sc solutions, the best separation is obtained using UTEVA resin. Therefore, for further experiments related to the labeling of DOTATATE, we chose this process.

### Radiolabeling and stability studies of DOTATATE conjugate

The DOTATATE was used as a model system for radiolabeling with the ^43^Sc radionuclide. High efficiency of labeling the DOTATATE with ^43^Sc was achieved as shown by the labeling yield exceeding 98 % for an amount of bioconjugate equal to or higher than 15 nmol (Table 5). The high yield showed that highly pure ^43^Sc was obtained after the separation process with UTEVA resin and is suitable for labeling biomolecules. When the reaction yield is not high enough, the labeled peptide can be easily purified using the Sep-Pak® C-18 column.

**Table 5:** Labeling yield as a function of the amount of DOTATATE used in the reaction

Amount of peptide [nmol]	Yield of labeling [%]
10	18.1 ± 1.0
15	99.2 ± 0.2
25	98.9 ± 0.2

The labeled DOTATATE radioconjugate exhibited high stability in human serum at 37 °C. After 14 h of incubation in the serum, more than 98 % of ^43^Sc remained in the radioconjugate.

In the present study, we found that synthesis of ^43^Sc-DOTATATE using cyclotron produced ^43^Sc could be adequate for nuclear medicine applications. Therefore, we believe that our method could be suitable for labeling different bioconjugates, regardless of whether they are other somatostatin analogs or a useful diagnostic peptide such as bombesin, substance P, or an oxytocin analog.

The procedure for labeling with ^43^Sc is as easy as that in the case of ^68^Ga and ^44^Sc which makes it possible to use commercially available kits. The 4 h half-life and obtainable GBq activities of ^43^Sc make possible the production and transport of the labeled bioconjugates to satellite PET centers, in analogy to ^18^F-FDG.

## Conclusions

The production of ^43^Sc in (α,n) and (α,p) nuclear reactions on a natural CaCO_3_ target was successfully performed, and extrapolation of the results obtained creates the opportunity to produce activity levels of ^43^Sc sufficient for medical applications. The ^43^Sc radionuclide has several advantages in comparison to the ^44^Sc recently proposed for PET imaging. Firstly, in contrast to ^44^Sc, it does not emit high-energy gamma rays that should be taken into consideration with regard to the radiation dose delivered to the patients and clinical staff. Emission of high-energy gamma rays also generates radiolytic decomposition of biomolecules, which is thought to be mediated by the formation of free radicals. This becomes an important issue when high quantities of radioactivity are used for labeling, as is necessary for clinical applications [26]. Also, co-production of the longer lived ^44m^Sc (*T*_1/2_ = 58.6 h) increases the radiation dose. Furthermore, in contrast to ^44^Sc, the production of ^43^Sc does not require an expensive highly enriched ^44^CaCO_3_ target, the price of which currently exceeds 14 USD/mg.

The proposed separation process of ^43^Sc from the calcium target is simple, reliable, efficient, and fast. Therefore, it is possible to use the same commercially available modular entity as that commonly used for preparation of ^68^Ga-radiopharmaceuticals. Unfortunately, availability of cyclotrons with high-current alpha beams is limited. Despite this, regional distribution following massive production at a single alpha facility is possible. We believe that the ^43^Sc obtained could be used instead of ^68^Ga in PET imaging and in planning peptide receptor radionuclide therapy with ^177^Lu- and ^90^Y-DOTA radiobioconjugates.

### Ethical approval

This article does not contain any studies with human participants or animals performed by any of the authors.
